# Together, We Inspire Smart Eating (WISE): An Examination of Implementation of a WISE Curriculum for Obesity Prevention in Children 3 to 7 Years

**DOI:** 10.1177/2333794X19869811

**Published:** 2019-08-12

**Authors:** Leanne Whiteside-Mansell, Taren Swindle, James P. Selig

**Affiliations:** 1 University of Arkansas for Medical Sciences, Little Rock, AR, USA

**Keywords:** Preschool, child care, nutrition, intervention

## Abstract

This study examined the implementation of a school-based, obesity prevention curriculum, Together, We Inspire Smart Eating (WISE), targeting 3- to 7-year-old low-income children. Survey data from a convenience sample were collected from educators and parents (N = 73, N = 188, respectively) at the beginning and end of a school year in which WISE was implemented. Educators also reported on lessons weekly. Measures to evaluate the success of the implementation were conceptually distinct implementation outcomes (Educators: Perceived Barriers, Appropriateness, Acceptability, Feasibility, Fidelity; Parents: Adoption, Appropriateness). WISE was successfully implemented in 33 target classrooms representing 7 preschool centers and 2 elementary schools. Based on educator report, perceived barriers were reduced. Educators rated Appropriateness, Acceptability, and Feasibility high. Evidence of Fidelity was mixed. Parents reported indicators of Adoption and Appropriateness high. The study provided support for WISE in preschools and elementary schools serving young children from low-resource homes.

## Introduction

Increasing consumption of fruits and vegetables (F/Vs) by young children is a target in efforts to combat obesity for several reasons.^1^ First, there is an established link between dietary intake and obesity.^2^ Unfortunately, many young children consume diets low in F/Vs.^2-6^ Second, interventions to improve eating habits in children have been effective. In particular, programs that include multiple components such as exposures to new foods, nutrition education for the adults delivering the intervention, and parent outreach components demonstrate impacts.^7^ Third, early intervention has long-term consequences on eating habits. Early exposure and consumption of F/Vs is linked to the consumption of these foods later in life.^8,9^

Families affected by poverty are disproportionally overweight and experience lower quality diets than their more affluent counterparts.^10^ Children from low-income families have less opportunity to experience a variety of F/Vs.^4^ Reflecting concern for the unique needs of low-income audiences, a US Department of Agriculture expert panel issued best practices recommendations for designing, delivering, and evaluating nutrition education for low-income audiences (eg, consideration of cost, literacy level).^11^ Children from low-income families benefit by federal programs in preschools and elementary schools when these recommendations are implemented.^12^

Many young children spend more than 30 hours in the care of programs such as Head Start, private child care, and family home day care.^13-15^ This has led to nutrition program standards for many of these programs that provide care and education for children.^16^ For example, Head Start is a US federal program targeting families in poverty that promotes school readiness for children from birth to age 5. Head Start programs vary in their structure and support families in a range of locations (eg, center-based, home-based). However, all Head Start programs adhere to performance standards requiring a 2-generational approach. That is, a 2-generational approach requires that the program provide services that support children and parents toward the goal of promoting healthy development of children.^17^ Regarding nutrition, Head Start performance standards include efforts to improve the home environment with parent education and to expose children to new and healthy foods at the center. Children over age 5 in the United States attend elementary schools—often for as many hours as childcare. Elementary schools in the United States provide meals for low-income children and are eligible for Fresh Fruit and Vegetable Program (FFVP) with different implementation standards. FFVP assists elementary schools to provide fresh F/Vs.^18^

The intervention, *Together, We Inspire Smart Eating* (WISE)^19-21^ is a curriculum-based intervention for low-income children aged 3 to 8 years in center-based or classroom settings in elementary school. WISE was developed to support educators’ implementation of nutrition best practice with the ultimate outcome of improving the young child’s consumption at home. In past studies, WISE outcomes related to educator knowledge gain^20^ and child consumption of the intervention have been evaluated. Specifically, WISE was evaluated in a quasi-experimental design with nonrandomized comparison classrooms.^19,20,22^ These studies showed that WISE training improved nutrition knowledge and behaviors of classroom educators.^22-24^ Children’s consumption of F/Vs at home reported by parents of children in WISE classrooms was higher than that reported by parents of children in non-WISE classrooms after 1 school year of exposure. A study report (in press) found the measure of skin carotenoids were consistent with parent reports. Based on classroom observations using a study-developed tool, Table Talk, teachers significantly reduced the use of inappropriate nutrition-related language (eg, “clean your plate”; “stop playing with your food”).^25^

Interventions, like WISE, targeting young children in school settings have the opportunity to improve the quality of children’s diet, target the most at-risk children, and educate a range of adults. However, to be effective, adult targets of intervention must perceive it to be suitable for their everyday use (ie, feasibility) and be satisfied with the characteristics of the intervention like content and delivery (ie, acceptability^26^). Feasible programs should have few implementation barriers. In fact, programs with low levels of feasibility and acceptability are not likely to be adopted widely, practiced with fidelity, or sustained in the long term.^26^ Increasingly, nutrition interventions are measuring feasibility and acceptability as implementation outcomes.^27,28^

The aim of this study is to report on aspects of the implementation evaluation of the WISE intervention. That is, in our other studies, we provide evidence that our implementation of WISE was useful in modifying the eating habits of children and classroom behavior of educators. In this study, we examine additional indicators that we expected to improve the likelihood that programs would adopt and sustain WISE. Specifically, the views of educators and parents about the intervention are likely to drive any expansion or long-term sustainability of WISE. Thus, we evaluated perspectives on the curriculum from both the view of parents and educators. Among educators, we used existing definitions of implementation outcomes as we well as WISE-specific potential barriers to assess appropriateness, acceptability, perceived fidelity, and barriers to implementation. Our focus with parents was similar but limited to measuring perceived adoption and appropriateness of WISE.

## Methods

This study was a substudy of a comprehensive study conducted in 3 phases. Phase I focused on the development of the WISE curriculum. A theoretical framework informed with an extensive review of research-based best practice and empirical study of target populations guided the development. In Phase II, and the target of this substudy, we focused on evaluation of parent and educator’s acceptance and their views on the usefulness of the WISE curriculum. Phase III expanded implementation with standardized training with evaluation focused on educator knowledge and attitude with 4 assessments from pretraining to end-school-year follow-ups to assess timing and stability of impacts.^20^

Researchers approached 2 Head Start agencies to collaborate on a multiphase study. Agencies were selected because they (*a*) had a history of successful research partnerships in the past, (*b*) served many children in multiple centers, and (*c*) operated in geographically different areas (one was urban and the other was rural). All pubic elementary schools located in the same rural city as one of the rural centers were recruited. Both agencies and all schools recruited agreed to participate. Within each school and Head Start centers all educators were recruited and participated. Parents of children that communicated in English or Spanish within each target classroom were recruited for interviews. Parent recruitment was stratified across classrooms in an effort to obtain interviews from parents from each classroom.

This study (Phase II) was conducted in 2 cohorts with a convenience sample over 2 years in 21 Head Start classrooms (n = 47 educators), 6 kindergarten classrooms, and 6 first grade classrooms (12 educators). One Head Start agency reported enrollment of 70% African American, 5% white, and 5% other/unknown families. The second agency enrollment included 18% African American, 70% white, and 13% other/mixed/unknown families. Elementary schools received FFVP and were demographically similar to the second Head Start agency. Parents (n = 278) and educators (n = 59) were interviewed at the beginning and end of the school year on a range of beliefs, practices, and attitudes about nutrition. Parents from 35 classrooms were interviewed and the number from each class ranged from 3 to 12, mean = 9, SD = 3.7, per class. The institutional review board at the University of Arkansas for Medical Sciences approved the study.

The intervention, *Together, We Inspire Smart Eating* (WISE)^19-21^ includes 3 components: (*a*) a classroom curriculum, (*b*) educator training, and (*c*) material/technology to educate parents. Differences in requirement and performance standards between preschool programs and elementary schools resulted in 2 implementation manuals and training agendas.^19,21^ For example, in elementary schools, FFVP required raw consumption with limited alterations (eg, limited dips allowed, but altering, cooking, or mixing foods is not allowed by FFVP policy).

### Classroom Curriculum

WISE is organized in an extensive manual with a chapter for each unit/month and an introductory chapter including best nutrition practice. Each unit/month focuses on 1 of 8 foods (eg, apples, bell peppers, tomatoes). The first week of each begins with a message from the farmer of the month and the arrival of the food into the classroom (Lessons 1 and 2). The curriculum mascot, Windy Wise, is a barn owl puppet who “travels” between the classroom and farm to deliver photos and letter updates on the growth of the food at the farm. Food is always introduced with sensory exploration to provide a consistent interaction with the food (Lesson 3).

The remainder of the month, educators select from a menu of food activities. Educators lead hands-on food preparation and cooking activities with children in small groups. The primary objective is to maximize children’s interaction with the target foods. Educators are provided with a suggested lesson plan schedule and suggestions for activities to integrate the material into other educational activities (eg, math, reading). Parent engagement materials, additional reading, and recipes for classroom food experiences are provided for each food. All recipes are budget-sensitive, healthy, and designed for children to complete the majority of the activities. Educators are provided a selection of closing activities to facilitate the transition to the next month. WISE foods from early in the school year often recur as companion ingredients in later WISE units and continue to be feature items on the menu for the entire school year.

### Educator Training

Training for preschool educators consists of an interactive 6-hour training based on adult learning theories. Changing adult behavior is thought to require both increase in knowledge and practice.^29,30^ Adult learners benefit from active rather than passive instruction, appropriate levels of challenge, monitoring and feedback, time to organize and integrate new ideas and concepts, and help generalizing to different contexts. Training content is related to participants’ own practice situations^31^ and utilize learners’ experiences as a point of departure.^30^ For early elementary educators, a training was conducted in 4 hours. Training in both groups included an overview of the role of educators and parents in child nutrition, exploration of the participant’s food attitudes and beliefs, practice using the WISE manual and implementation process, and guided planning on connections with families.

### Parent Engagement

Each month, parents received “back pack” letters from the farmer to introduce the target food. Volunteering classrooms established a restricted, classroom-based Facebook groups creating an opportunity for parent-to-parent and educator-to-parent conversations.^32^ Monthly parent-night meeting materials were available to allow parents to experience a new food and participate in preparing a healthy snack.

### Measures

Measures to evaluate the success of the implementation were organized based on conceptually distinct implementation outcomes: Perceived Barriers, Appropriateness, Acceptability, Feasibility, Fidelity.^26^ Assessment was based on educator and parent interviews pre- and post-implementation and educator assessments after individual lessons. Educators reported on 12 items assessing self-efficacy regarding classroom nutritional training on a 4-point scale (1 = Not at all confident) with higher scores indicating more self-efficacy.^33,34^ Educators reported on 5 potential barriers to nutrition education (1 = yes, 0 = no) and on the quality of the WISE food experience on a 5-point scale (1 = Strongly agree). Strongly and agree ratings were combined to examine percent agreement.

Educators rated the fidelity of implementation of each lesson. Items were summarized and combined across educator across the school year. Responses were coded to indicate educator endorsement on a binary (1 = yes, 0 = no) or a Likert-type scale (1 = Not at all to 4 = Very much).

Parents rated the frequency of WISE-related behaviors (1 = never to 6 = every day) to indicate adoption. Parents rated Appropriateness statements about the WISE curriculum on a 5-point scale (1 = Strongly agree).

## Results

All elementary school educators had at least a BA degree, and 50% of Head Start teachers had a BA (3 had high school degree; and 14 had associate degrees). Most were white (55.6%); 34% were African American; and 4 were Hispanic. Parents did not provide demographic information.

As seen in Table 1, Perceived Barriers for educators decreased for 2 of the 6 areas examined across the school year. Educators reported feeling more confident and being sufficiently trained by the end of the year. For example, “lack of training” was reported significantly less as a barrier by the end of the school year (52% before WISE and 29% after χ^2^[1, N = 48] = 13.17, *P* < .01). Teacher self-efficacy significantly improved (*t*[47] = 3.32, *P* < .002, Cohen’s *d* = .34).

**Table 1.** Educator Reports on Barriers, Appropriateness, Acceptability, and Fidelity of WISE.

	Fall, Mean (SD) or %	Ratings of Lessons (%)	Spring, Mean (SD) or %
Perceived barriers	Percent		Percent
I understand the goal of WISE^a^	—		100%
Lack of training/skill^a,**^	52%		29%
Lack of equipment^a^	21%		10%
Rules do not allow^a^	2%		6%
Parents do not cooperate^a^	19%		35%
Lack of director/agency support^a^	6%		8%
Nutrition self-efficacy (12 items, α = .93)^b,**^	2.94 (0.51)		3.12 (0.45)
Appropriateness^c^			
WISE made a difference in what the children ate at school.			100%
WISE made a difference in what the children ate at home.			65%
WISE made a difference for families.			73%
WISE made my food experiences easier.			87%
Acceptability			
I enjoyed WISE.^c^			94%
I think children enjoyed WISE.^c^ (this lesson)^d,e^		97%	98%
Percent of children that tried food/lessons^d^		93%	
The children were eager to try the food.^d,e^		96%	
I hope my program adopts WISE next year.^c^			79%
I would recommend WISE.^c^ (this lesson)^d,e^		97%	88%
Feasibility			
I am comfortable doing lessons.^c^			96%
WISE is too much extra work.^c^			12%
WISE improved my food experiences.^c^			88%
The lesson was successful.^c^		97%	
Fidelity			
I tried the food.^d,f^		59%	
Windy WISE visited during or after our lesson.^d,e^		80%	
I did the lesson with groups of 3 to 6 children at a time.^d,f^		57%	
I had conversations with the children about food during the lesson.^d,f^		44%	
I emphasized trying the food (not liking).^d,f^		25%	
I made positive comments about the food.^d,f^		43%	
I encouraged children to talk with their parents about the lesson.^d,f^		43%	
Indirect evidence of fidelity^e,g^			
The children had an opportunity to use math skills. (2 items)		91%	
I introduced new vocabulary. (3 items)		47%	

Abbreviation: WISE, We Inspire Smart Eating.

a Response Yes or No, *N* = 52.

b Four-point scale (1 = Not at all confident), *N* = 52.

c Five-point scale (with percent of Strongly Agree and Agree displayed), *N* = 52.

d Reports from 35 educators with *N* reports = 416-453.

e Endorsed = “Very much” or “Quite a bit.”

f Endorsed = “Yes.”

g Fidelity, Extension to Academic Activities only assessed by cohort 2 educators, *N* = 323-325.

** *P* < .00.

Child and educator Acceptance was high (Table 1). After a year of implementation, educators reporting enjoying WISE (94%) and thinking children also enjoyed it (98%). Educators reported that they would recommend most lessons (97%) and the curriculum as a whole (88%). Educators reported children tried the target food (93%) and were enthusiastic about the lesson (96%).

Feasibility was supported with high ratings of comfort (96%) and success of lessons (97%). WISE was not perceived as too much extra work; only 12% reported it as extra work, while most (88%) reported it as an improvement to food experiences.

Educators’ rating of behaviors included in the WISE curriculum shows only 3 behaviors were reported at least 50% of the time by educators (Ratings of Lessons in Table 1). That is, educators reported they were most successful with these 3 behaviors. For example, most educators reported including the curriculum mascot, Windy WISE, in most lessons (80%). Educators reported they tried the WISE food (59%) and conducted the lesson in small groups (57%). However, most educators reported a focus on children liking the food instead of just trying the food. In educational settings, curriculum that build on other education activities are thought to be more feasible and valued to implement.^35^ Most educators included math skills in most lessons (91%).

As shown in Table 2, parents reported behaviors that suggested adoption of WISE. For example, there was a signficiant increase in buying fruits or vegetables because a child asked. Parents, like educators, had high ratings of Appropriateness; 77% endorsed “WISE made a difference in what my child eats.” Parents reported WISE as a factor in changes in food consumption and purchases at home.

**Table 2.** Parent Support of WISE: Adoption and Appropriateness.

	Fall, Mean (SD)	Spring, Mean (SD)
Parent Adoption		
Tell me how often you^a^		
Visit the classroom Facebook page	2.03 (1.71)	2.28 (1.75)
Attend or volunteer at a food experience at the center	1.16 (.57)	1.44 (0.89)*
Buy fruits or vegetables because your child asks for them	3.49 (1.49)	3.60 (1.23)*
Parent Appropriateness		Percent agree
I understand the goal of WISE		94%
Thinking more about the WISE program^b^		
WISE made a difference in what my child eats		77%
My child ask me to buy WISE foods		71%

Abbreviation: WISE, We Inspire Smart Eating.

a N ranges from 198 to 277; response options ranged from 1 (never) to 6 (everyday).

b N ranges from 198 to 199; response collapses Strongly agree and Agree.

* *P* < .05.

## Discussion

Effective programs to improve diet and health outcomes must be perceived as practical and useful. This study provided evidence of a well-recieved implementaiton of WISE by educators and parents of the research-based, obesity prevention curriculum and training, WISE. In this study, we targeted preschool programs and elementary schools that served a high percent of children from low-income families. Furthermore, this study provided an example of assessing indicators of feasibility and acceptability at multiple time points and from multiple perspectives include parent and educator.

This study provides an objective and systematic approach to the study of indicators of intervention feasibility and acceptability. Recent work in a similar setting used focus group interviews and post-intervention surveys with parents and educators to explore feasibility and acceptability.^27^ As in our study, findings indicated high levels of both constructs. These studies provide evidence that the preschool setting can accommodate nutrition interventions when they are designed with integration in mind.

This study provided evidence that parents found WISE to be acceptable and that parents perceived changes in child consumption because of WISE. This is a promising finding in light of research documenting the barriers of early childhood educators in engaging parents around nutrition topics.^36,37^ Parents linked the child’s experience in WISE with child requests in food purchases. They reported an increase in purchasing F/Vs because the child asked for them. This suggests that WISE was effective in leveraging “pester power” for increasing purchase of healthy, rather than unhealthy, snacks.^38,39^

The curriculum was designed to avoid increasing the burden on educators. WISE was integrated into existing program requirements without having to set aside specific time to implement “nutrition education.” That is, WISE structured the time already established in most programs and set aside for food experiences or center time activities. Likely because training was included in the WISE curriculum, fewer educators indicated lack of training as a barrier. Although the increase was not significant, at the end of the school year an increase of 4% of educators identified rules as a barrier. Furthermore, at the end of the year an additional 16% of educators reported “parent involvement” as barriers presumably meaning their lack of involvement. The reason for this trend is not clear; however, it may be that educators became aware of rules they had not known previously. In addition, as educators focus on nutrition, they may begin to notice parent engagement (or lack of it) around nutrition and realized it was a barrier.

Limitations to the study include self-report data and study-developed measures. Self-report data have inherent weakness where social desirability is a concern as may be the case in this study. Self-report of educator best practice increased from fall to spring. However, many educators did not routinely report the use of some key early childhood best practice. For example, educators did not report frequent use of the recommendation to emphasize trying (instead of liking) food. The lack of self-report of this practice may be evidence that educators were not driven by socially acceptable answers. Future studies of WISE should include a full-randomized control study.

Implementation science is a quickly evolving discipline and, unfortunately, when the developers of this study selected measures, no validated measures of the implementation constructs were available. The study developers create tailored measures based on the conceptual definitions of key implementation outcomes in the field at the time. Since that time, pragmatic measures that are adaptable to multiple interventions have been developed, and their use is recommended.^40^

## Implications for Research and Practice

WISE is based on an extensive research foundation and grounding in best practices from a comprehensive array of relevant literature (ie, obesity prevention, early childhood, and adult learning styles). WISE was developed to be integrated into existing standards of programs serving high-risk children. This study provided promising support for the dissemination of a research-based intervention.
